# Mental health status, burden and satisfaction with social support in the portuguese family caregivers of people with mental disorders: a cross-sectional study

**DOI:** 10.1192/j.eurpsy.2025.1603

**Published:** 2025-08-26

**Authors:** C. Laranjeira

**Affiliations:** 1 School of Health Sciences; 2 ciTechCare, Polytechnic Institute of Leiria, Leiria, Portugal

## Abstract

**Introduction:**

Current political recommendations on mental health aim to keep people with mental illness in the community, emphasizing the need to develop a natural social support network, which includes families, giving rise to the role of the family caregiver.

**Objectives:**

The aims of the study were: a) to identify sociodemographic variables that influence the anxiety, depression and stress of the caregiver of people with mental disorders; b) to analyze the relationship between social support and caregiver burden with anxiety, depression and caregiver stress.

**Methods:**

We carried out a cross-sectional, descriptive-correlational study, with 274 portuguese caregivers who were recruited using a convenience sampling technique. The instruments used were the Satisfaction with Social Support Scale (ESSS); Zarit Burden Interview Scale and Depression Anxiety Stress Scales (DASS-21). Descriptive statistics and nonparametric tests were used as required. Data were analyzed using SPSS-22.0. The protocol was approved by local ethical committee.

**Results:**

Most of participants were female (67.0%) and aged between 26 and 85 years old, with an average of 66.13 (SD=5.61) years old. Female caregivers have higher rates (p<0.05) of anxiety, depression and stress. Caregivers who lived in rural areas have higher levels of depression (p=0.036) and stress (p=0.029). On the other hand, the greater the perceived overload, the higher the levels of anxiety (p<0.001), depression and stress (p<0.001).

**Conclusions:**

The results suggest the need to invest in local and community intervention strategies to promote mental health and prevent mental illnesses. In this vein, health institutions must support educational and monitoring measures that screen mental illness situations early.

**Disclosure of Interest:**

None Declared

